# BRCA1-deficient breast cancer cell lines are resistant to MEK inhibitors and show distinct sensitivities to 6-thioguanine

**DOI:** 10.1038/srep28217

**Published:** 2016-06-17

**Authors:** Yuexi Gu, Mikko Helenius, Kristiina Väänänen, Daria Bulanova, Jani Saarela, Anna Sokolenko, John Martens, Evgeny Imyanitov, Sergey Kuznetsov

**Affiliations:** 1 Institute for Molecular Medicine Finland (FIMM), University of Helsinki, PO Box 20, FIN-00014, Helsinki, Finland; 2 Department of Biology, University of Eastern Finland, PO Box 111, FI-80101 Joensuu, Finland; 3 Department of Tumor Growth Biology, N.N. Petrov Institute of Oncology, St.-Petersburg 197758, Russia; 4 Department of Medical Genetics, St.-Petersburg Pediatric Medical University, St.-Petersburg 194100, Russia; 5 Erasmus University Medical Center, Daniel den Hoed Cancer Center, Department of Medical Oncology and Cancer Genomics Center, Dr. Molewaterplein 50, 3015 GE Rotterdam, the Netherlands; 6 Department of Oncology, I.I. Mechnikov North-Western Medical University, St.-Petersburg 191015, Russia

## Abstract

Germ-line or somatic inactivation of BRCA1 is a defining feature for a portion of human breast cancers. Here we evaluated the anti-proliferative activity of 198 FDA-approved and experimental drugs against four BRCA1-mutant (HCC1937, MDA-MB-436, SUM1315MO2, and SUM149PT) and four BRCA1-wild-type (MDA-MB-231, SUM229PE, MCF10A, and MCF7) breast cancer cell lines. We found that all BRCA1-mutant cell lines were insensitive to inhibitors of mitogen-activated protein kinase kinase 1 and 2 (MEK1/2) Selumetinib and Pimasertib in contrast to BRCA1-wildtype control cell lines. However, unexpectedly, only two BRCA1-mutant cell lines, HCC1937 and MDA-MB-436, were hypersensitive to a nucleotide analogue 6-thioguanine (6-TG). SUM149PT cells readily formed radiation-induced RAD51-positive nuclear foci indicating a functional homologous recombination, which may explain their resistance to 6-TG. However, the reason underlying 6-TG resistance of SUM1315MO2 cells remains unclear. Our data reveal a remarkable heterogeneity among BRCA1-mutant cell lines and provide a reference for future studies.

Germ-line mutations in BRCA1 are associated with about 25% of familial breast and ovarian cancers, while somatic inactivation of BRCA1 is observed in up to 5% of sporadic breast cancers^1^,^2^. The best-known function of BRCA1 is associated with its role in repair of DNA damage, particularly of double-stranded DNA breaks (DSBs), one of the most severe types of DNA lesions^3^. BRCA1 is recruited to sites of DNA damage, where it serves as a binding scaffold for other DNA repair proteins^4^,^5^, ultimately facilitating the loading of RAD51 recombinase to sites of DNA damage and, thus, enabling the homologous recombination-mediated (HR) DNA repair^3^. In addition, BRCA1 interacts with BARD1 protein via its N-terminal RING domain forming a heterodimer with an E3 ubiquitin ligase activity^6^. BRCA1-BARD1 heterodimer ubiquitinates multiple target proteins, including CtIP (RBBP8), nucleophosmin (NPM1, B23), claspin (CLSPN), and others, thus affecting DNA repair-related and -unrelated signaling^7^,^8^,^9^,^10^.

BRCA1-deficient breast cancers are four to eight time less likely to express estrogen alpha receptor (ERα) compared with sporadic breast cancers of similar grade, and tend to lack expression of progesterone, and ErbB2 receptors, that formally defines them as triple-negative tumors^11^. Compared with sporadic tumors, BRCA1-deficient breast cancers are also five to ten times more likely to express cytokeratins 5/6 (CK5/6) associated with the mammary basal (myoepithelial) cells^11^. Such tumors also tend to be genomically unstable^12^ and reportedly contain higher fraction of cells with cancer stem cell properties^13^,^14^, which together makes up for a highly aggressive tumor phenotype. This is surprising considering that a complete loss of Brca1 is early embryonic lethal^15^,^16^, and suppression of BRCA1 in primary cells and even established cancer cell lines has a growth-suppressive effect, similar to defects in other HR genes^17^,^18^,^19^. Nevertheless, at least four BRCA1-mutant breast cancer cell lines were successfully established: HCC1937, MDA-MB-436, SUM1315MO2, and SUM149PT^20^,^21^,^22^,^23^. These cell lines carry distinct protein-truncating mutations in one allele of BRCA1, while the other allele is lost^23^. All of them have a mutant TP53 and express the triple-negative basal-like phenotype like typical BRCA1-mutant breast cancers^23^. These cell lines are widely used to study various functional aspects of BRCA1, often in comparison with isogenic cells expressing wild-type BRCA1 cDNA^24^.

Here we sought to investigate common and distinct features for BRCA1-mutant cell lines in comparison with a panel of BRCA1-proficient cell lines. We tested growth-inhibiting effects of 198 FDA-approved and experimental drugs on four BRCA1-mutant and four comparable BRCA1-wild-type human breast cancer cell lines. We found that all BRCA1-mutant cell lines were relatively resistant to MEK1/2 inhibitors. In addition, two of the four BRCA1-mutant cell lines were hypersensitive to 6-thioguanine (6-TG) consistent with earlier reports predicting such effect for HR-deficient cells. We also found possible explanations for resistance of two other BRCA1-mutant cell lines to 6-TG, thus providing support to this gene-drug interaction.

## Results

### BRCA1-mutant cell lines are resistant to inhibitors of MEK1/2

**Figure 1:**
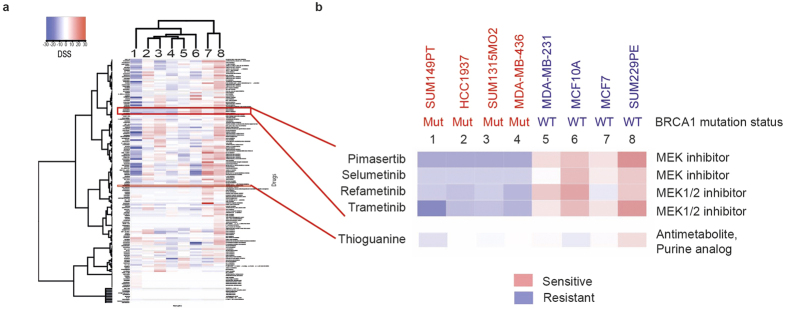
A high throughput chemical screen reveals resistance of BRCA1-mutant breast cancer cell lines to MEK inhibitors. (**a**) A library of 198 FDA-approved and experimental drugs was tested against a panel of BRCA1-mutant (1–4) and BRCA1-wild-type (5–8) cell lines. Relative cell viability was measured using CellTiter-Blue fluorescent metabolic assay after three days of incubation and presented as a heat map of differential drug sensitivity scores (dDSS). (**b**) Differential DSS scores for MEK1/2 inhibitors and 6-thioguanine are highlighted. Cell line names are shown above. Mut, BRCA1 mutant; WT, BRCA1 wild type.

High-throughput small molecule screening has been widely used to identify pharmacologically-relevant compounds targeting cancer cells with specific genetic abnormalities. We used a locally available library of 198 FDA-approved drugs or targeted experimental drugs^25^ to investigate pharmacological vulnerabilities of four human cells carrying a mutant BRCA1 – HCC1937, MDA-MB-436, SUM1315MO2, and SUM149PT^20^,^22^,^23^. The cell line identity was verified using microsatellite markers, and signature BRCA1 mutations were confirmed by Sanger sequencing (Supplementary Figure S1). The following cell lines were used as BRCA1 wild type controls: MDA-MB-231 and SUM229PE (basal-like, p53-mutant), MCF10A (basal-like, p53 wild type), and MCF7 (luminal, p53 wild type)^23^,^26^. The screening was performed as described in Materials and Methods. Drug Sensitivity Scores (DSS)^25^ calculating the area under the dose response curve, relative to the total area between 10% threshold and 100% inhibition, further normalized by a logarithm of the top response^27^. The final data are shown as a differential DSS (dDSS) representing a Z-score from an average DSS value for all cell lines for each drug. (Fig. 1 and Supplementary Table S1).

**Figure 2:**
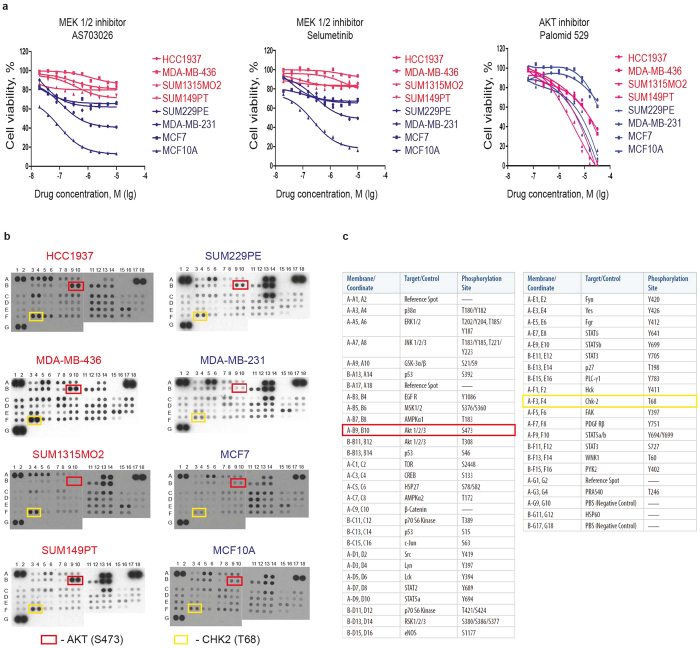
BRCA1-mutant breast cancer cell lines are resistant to MEK1/2 inhibitors. (**a**) Sensitivity of BRCA1-mutant (red) and BRCA1-wild-type (blue) cell lines to MEK1/2 and Akt inhibitors was re-tested in 96-well plates using CellTiter-Blue fluorescent metabolic assay. Each data point represents an average of four replicas. Non-linear regression curves were calculated using GraphPad Prism. (**b**) to When compared with BRCA1-wild-type cell lines (on the right), BRCA1-mutant cell lines (on the left) have increased phosphorylation of AKT (except SUM1315MO2) and CHK2 kinase as revealed with a Human Phospho-Kinase Antibody Array. Each kinase is tested in duplicates. Spots corresponding to AKT phosphorylated at S473 and Chk2 phosphorylated at T68 are highlighted with red and yellow rectangles, respectively. Spot coordinates are marked along the left and the top edges of each membrane. (**c**) List of all targets of the Human Phospho-Kinase Antibody Array and their coordinates.

Surprisingly, only one class of small molecule compounds demonstrated a consistent pattern of growth inhibition effect differentiating between all BRCA1-wildtype as opposed to BRCA1-mutant cell lines. All four BRCA1-mutant cell lines were relatively resistant to four inhibitors of Mitogen-Activated Protein Kinase Kinase (MAPKK, also known as MAP2K and MEK)-selumetinib (AZD6244), trametinib (GSK1120212), refametinib (RDEA119) and pimasertib (AS-703026) (Fig. 1). This result was validated by testing the sensitivity of the same cell lines to pimasertib and selumetinib at ten different concentrations in 96-well plates (Fig. 2a).

MEK is an important kinase within the ERK mitogenic signaling pathway^28^. Sensitivity to MEK inhibitors is often associated with activating mutations in the upstream elements of this pathway such as Ras or Raf oncogenes^29^,^30^,^31^. None of the BRCA1-mutant cell lines harbor such mutations, which could explain their relative resistance to MEK inhibitors. However, while one of the control cell lines MDA-MB-231 harboring mutant KRAS and BRAF^26^ indeed shows a relatively steep sensitivity to MEK inhibitors, SUM229PE cells carrying a mutant KRAS gene are noticeably less sensitive (Fig. 2a), suggesting that additional mechanisms could determine the sensitivity. We hypothesized that other proliferative signaling pathways could mediate the low sensitivity of BRCA1-mutant cell lines to MEK inhibitors. To test this, we measured activities of all essential signaling kinases in these cell lines using a phospho-kinase antibody array (Fig. 2b). We found that three out of four BRCA1-mutant cell lines (except SUM1315MO2) had an increased phosphorylation of AKT/PKB protein kinase at Ser473 residue when compared to BRCA1 wild-type control cell lines (Fig. 2b). AKT is a serine-threonine protein kinase regulating many intracellular processes including cell survival^32^. Consistent with its primary pro-survival function in BRCA1-mutant cells, these cells tend to be more sensitive to AKT inhibitor palomid 529 (Fig. 2a), which mostly correlates with increased PARP cleavage (Supplementary Figure 2), although this feature does not clearly separate BRCA1-mutant from BRCA1-wild type cells. On the other hand, all BRCA1-mutant cell lines also revealed higher phosphorylation of the cell cycle checkpoint kinase CHK2 at Thr68 compared to control cells, which possibly indicates a higher checkpoint activity and, therefore, improved survival. Thus, the observed resistance of BRCA1-mutant cell lines to MEK inhibitors may be associated with AKT- and CHK2-mediated cellular survival.

### Distinct responses of BRCA1-mutant cells to thiopurines

**Figure 3:**
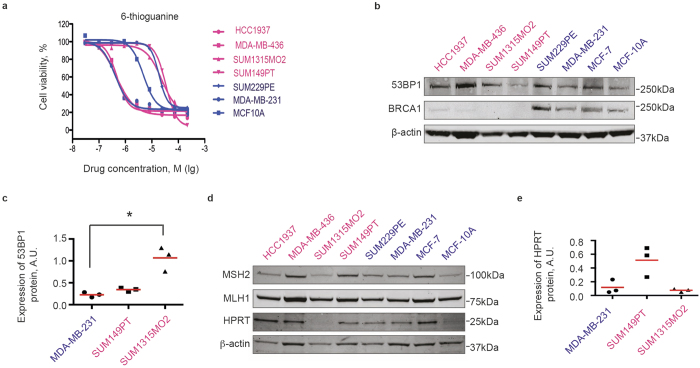
BRCA1-mutant cell lines show distinct sensitivities to 6-thoguanine. (**a**) Sensitivity of BRCA1-mutant (red) and BRCA1-wild-type (blue) cell lines to 6-thioguanine was tested in 96-well plates in four replicas using CellTiter-Blue metabolic assay. Each data point represents an average of four replicas. Non-linear regression curves were calculated using GraphPad Prism. (**b**) Western blot demonstrating that BRCA1-mutant (red) cell lines express full-length 53BP1 protein, but not BRCA1. (**c**) Quantification of 53BP1 protein expression for select cell lines. Individual normalized band intensity values in arbitrary units (A.U.) from three independent experiments are shown. Red lines show average values for each cell line. Statistical significance was evaluated with the Dunn’s multiple comparisons test following a significant non-parametric Kruskal-Wallis test using GraphPad Prism software. *p <* 0,05*. (**d**) Western blot reveals expression of mismatch repair proteins MSH2 and MLH1 in all cells, but lack of the HPRT protein in SUM1315MO2 cell line, likely explaining its resistance to 6-thioguanine. β-actin is used as loading control. (**e**) Quantification of HPRT protein expression for select cell lines as described in (**c**).

Recently, 6-thioguanine (6-TG) was found to selectively kill BRCA2-deficient cells due to its essential role in HR, which is required during mismatch repair^33^. Since BRCA1, similarly to BRCA2, is highly important for HR, we asked whether 6-TG would have a selective toxicity against BRCA1-deficient cells. However, the test results did not reveal any obvious correlation between the BRCA1 mutation status and sensitivity to 6-TG. Two BRCA1-mutant cell lines SUM1315MO2 and SUM149PT, and one control cell line SUM229PE were resistant to 6-TG with IC50 around 50 μM (Fig. 3a). Two other BRCA1-mutant cell lines HCC1937 and MDA-MB-436, and one control cell line MDA-MB-231 were sensitive to 6-TG with IC50 less than 1 μM (Fig. 3a). Non-malignant mammary epithelial cell line MCF10A demonstrated an intermediate sensitivity to 6-TG with IC50 about 10 μM (Fig. 3a).

**Figure 4:**
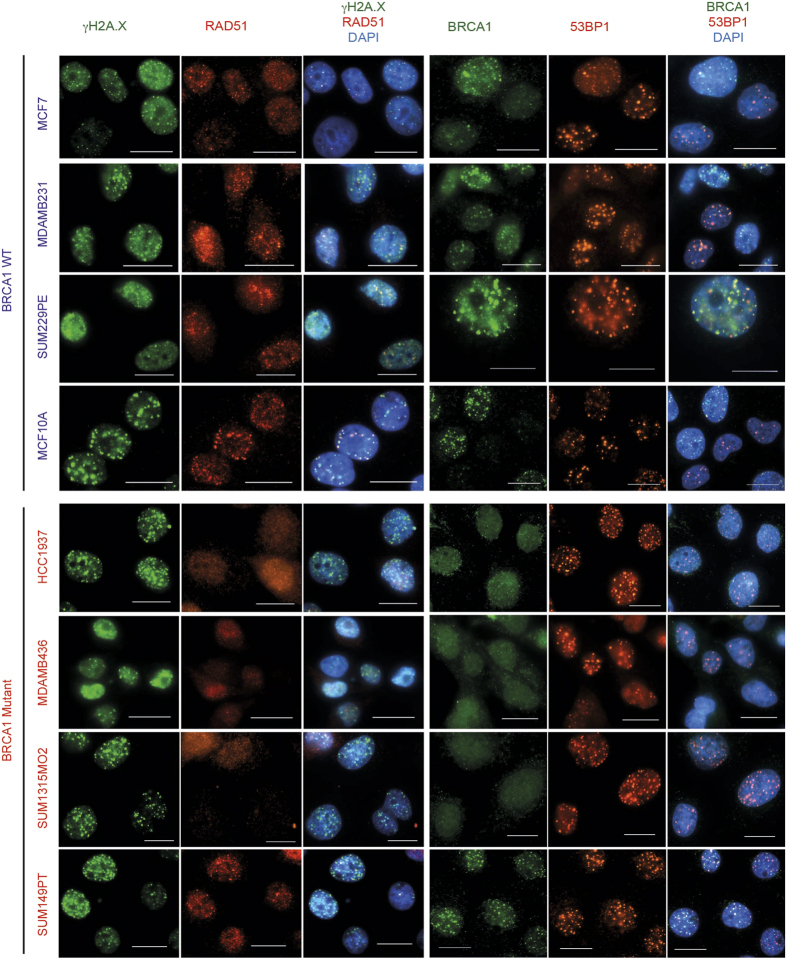
Homologous recombination proficiency in cell lines used in the study. Cells grown on coverslips were irradiated with 5 Gy and formation of nuclear DNA repair foci was evaluated after 6 hours using immunofluorescence. All BRCA1-wild-type cell lines readily form RAD51 foci colocalizing with γH2AX foci or BRCA1 foci colocalizing with 53BP1 foci indicating functional homologous recombination. Notice that SUM149PT cells also form nuclear foci despite mutant BRCA1. Scale bars represent 20 μm.

**Figure 5:**
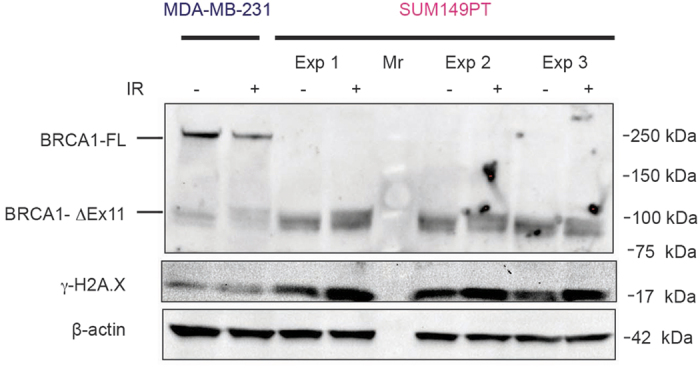
SUM149PT cells express a truncated partially functional BRCA1 protein. Cells were irradiated with 6 Gy and lysed after 6 hours for analysis by Western blotting. Data for three independent experiments are shown (Exp 1–3). Note the band shifting after irradiation probably reflecting changes in the phosphorylation status. Mr, molecular weight marker; BRCA1-FL, full-length BRCA1 protein; BRCA1-ΔEx11, truncated BRCA1 protein lacking exon 11.

First, to explain this result, we re-evaluated the expression of BRCA1 and the HR proficiency of all cell lines. Western blot analysis did not reveal a full-length BRCA1 protein in BRCA1-mutated cell lines in contrast to control cells (Fig. 3c). In addition, no secondary mutations restoring the open reading frame of BRCA1 was found by sequencing BRCA1 locus (Supplementary Figure S1 and data not shown). However, while control cell lines readily formed BRCA1 nuclear foci 6 hours after ionizing irradiation, HCC1937, MDA-MB-436, and SUM1315MO2 cells formed neither BRCA1, nor RAD51 DNA repair nuclear foci under the same conditions (Fig. 4), indicating a defective homologous recombination process. The only exception was the SUM149PT cell line, which, despite undetectable full-length BRCA1 protein on a Western blot, revealed radiation-induced BRCA1 and RAD51 nuclear foci by immunofluorescence (Fig. 4). Instead of the full-length BRCA1 protein around 220 kDa in size, SUM149PT cells strongly express a short splice-variant around 100 kDa (Fig. 5) that, reportedly, lacks exon 11^34^, yet accumulates in nuclear radiation-induced foci (Fig. 4) and, therefore, may be partially functional. 53BP1 protein, whose loss can restore HR in the absence of the functional BRCA1, was readily detectable both by Western blot (Fig. 3b,c) and by immunofluorescence in all cell lines tested (Fig. 4) suggesting that this mechanism is inapplicable in improved survival of these particular cell lines. Thus, HR proficiency of the SUM149PT cell line may be responsible for its resistance to 6-TG. However, a different mechanism is at play for BRCA1-deficient SUM1315MO2 and control MDA-MB-231 cell lines.

Alternatively, resistance to 6-TG can be mediated by mutations in essential mismatch repair proteins MLH1, MSH2, MSH6, and PMS1, which are required to recognize mismatched nucleotide pairs^35^. Such mutations also lead to microsatellite instability^36^. However, microsatellite instability has never been reported for any of the BRCA1-mutant cell lines^26^. In addition, we were able to detect expression of the full length MLH1 and MSH2 proteins by Western blot in all cell lines that we tested suggesting that the diverse sensitivity of BRCA1-mutant cell lines to 6-TG is not associated with defective mismatch repair (Fig. 3d). On the other hand, resistance to 6-TG has been associated with a loss of the enzyme hypoxanthine-guanosyl phosphoribosyl transferase (HGPRT or HPRT) mediating its bioactivation by converting 6-TG into 6-thioguanine monophosphate, which then can be incorporated into RNA or DNA^37^. Our initial data indicated that SUM1315MO2, one of the most resistant cell lines, expressed very low levels of the HPRT protein (Fig. 3d), which might explain the resistance to 6-TG. However, a careful quantification of the HPRT protein level from three independent experiments failed to demonstrate a significant difference between SUM1315MO2 and other cell lines (Fig. 3e) ruling HPRT out as a reason for its 6-TG resistance. Together, our data provide a plausible explanation for observed sensitivities to 6-TG for all tested cell lines except MDA-MB-231 and SUM1315MO2.

## Discussion

Breast cancer cell lines are useful to investigate molecular mechanisms of breast cancer formation and identify potential therapeutics strategies. We performed a comprehensive testing of a panel of BRCA1-mutant and comparable BRCA1-wild-type breast cancer cell lines for sensitivity to 198 approved and investigational drugs to identify common vulnerabilities. Our screen revealed a remarkable heterogeneity among the four BRCA1-mutant breast cancer cell lines in their responses to most drugs. The only feature common for all four BRCA1-mutant cell lines is resistance to MEK inhibitors. On the one hand, this is consistent with an earlier finding that BRCA1-mutant breast cancer cell lines do not usually harbor activating mutations in RAS and BRAF oncogenes^26^, which normally make cells susceptible to MEK inhibitors^29^,^30^,^31^. However, at least two out of four control cell lines, MCF7 and MCF10A, do not carry mutations in this pathway, but yet are sensitive to MEK inhibitors. On the other hand, we found that three BRCA1-mutant cell lines, except SUM1315MO2, had an increased phosphorylation of AKT at Ser437, which might be responsible for a better cell growth when the MAPK-ERK pathway was inhibited with MEK inhibitors^38^. Indeed, BRCA1-mutant cells revealed a tendency for a higher sensitivity to AKT inhibitors. Moreover, BRCA1 has been shown to bind phosphorylated AKT targeting it for ubiquitin-mediated degradation^39^, and, thus, functioning as a negative regulator of the AKT pathway. However, SUM1315MO2 cells do not show any appreciable phosphorylation of AKT, yet in terms of sensitivity to MEK inhibitors, they clearly cluster with other BRCA1-mutant cell lines suggesting that AKT phosphorylation may not fully explain the observed phenotype. A third explanation, however, may be associated with an increased phosphorylation of CHK2 kinase in all BRCA1-mutant cell lines relative to four control cell lines. CHK2 regulates an ATM-mediated cell cycle checkpoint in response to DNA damage^40^. It is tempting to speculate that CHK2-mediated cell cycle checkpoint becomes activated in BRCA1-mutant cells due to HR defect. Therefore, a therapeutic potential of CHK2 and AKT inhibitors, separately or in combination, may be promising to explore.

Apart from such resistance to MEK inhibitors, BRCA1-mutant cell lines did not demonstrate any common drug vulnerabilities in our assay. Considering an earlier demonstration of sensitivity of HR-deficient cells to thiopurines, we investigated whether BRCA1 deficiency produced a similar effect. Somewhat unexpectedly, our screen revealed that two BRCA1-mutant cell lines were as resistant to 6-TG as a BRCA1-wild-type SUM229PE cell line, while two other BRCA1-mutant cell lines were almost 100-times more sensitive to 6-TG. Resistance of SUM149PT cells to 6-TG may be explained by its HR-proficiency while the reason behind SUM1315MO2 resistance remains unclear. Sensitivity of HCC1937 and MDA-MB436 cell lines to 6-TG is consistent with their HR-deficiency and mutant BRCA1 status, thus supporting the initial prediction. This result appears to contradict the study by Kinsella and colleagues^41^ demonstrating that HCC1937 cells are more resistant to 6-TG than isogenic cells expressing wild-type BRCA1 cDNA. However, the IC_50_ value for isogenic BRCA1-transfected HCC1937 cells in this study is less than two-fold lower than that of the original cells, and both of them are within the micromolar range similar to our hypersensitive cell lines including HCC1937, and two orders of magnitude lower than truly 6-TG resistant cell lines. In addition, our similar screen on MDA-MB-231 cell line, which is rather sensitive to 6-TG to begin with, indicates that depletion of BRCA1 with siRNAs may further increase their sensitivity to 6-TG^37^. Thus, our data suggest that 6-TG may be indeed a promising therapeutic agent against BRCA1-deficient cancers provided that cancer cells are HR-deficient and have a functional mismatch repair machinery.

Taken together, our data provide a comprehensive description of drug vulnerabilities of existing BRCA1-mutant breast cancer cell lines. However, the output of the assay was growth inhibition, while measurement of cytotoxicity, apoptosis induction, or expression of various reporters as assay readout could produce different results^42^. Nevertheless, the results reveal a remarkable heterogeneity among BRCA1-mutant cell lines, which should be taken into consideration for future studies.

## Materials and Methods

### Cell culture

Breast cancer cell lines HCC1937, MDA-MB-231, MDA-MB-436, MCF-7, and the non-malignant MCF10A cells were purchased from the American Type Culture Collection (ATCC). All SUM cell lines were kindly provided by Dr. John Martens (Erasmus University Medical Center, the Netherlands). HCC1937 cells were grown in RPMI 1640, MDA-MB-231, MDA-MB-436, and MCF-7 in DMEM, supplemented with 10% fetal bovine serum (FBS), 2 mM L-glutamine and 1 × Penicillin-Streptomycin (Life Technologies). MCF-10A cells were cultivated in DMEM/F-12 medium supplemented with 5% horse serum, 20 ng/ml EGF, 0.5 μg/ml hydrocortisone, 0.1 μg/ml cholera toxin, 10 μg/ml insulin and 1 × Penicillin-Streptomycin. SUM cell lines were all grown in Ham’s F-12 medium containing 5% FBS, 10 μg/ml insulin, 1 × Penicillin-Streptomycin (Life Technologies), and supplemented either with 10 ng/ml EGF (for SUM1315MO2), or with 0.5 μg/ml hydrocortisone (for SUM149PT and SUM229PE). Cells were propagated at 37 °C in a 5% CO_2_ atmosphere. The cell identity was confirmed at the FIMM technology center according to ATCC guidelines using the Promega StemElite ID System.

### Drug sensitivity testing

The screening was performed essentially as described by Pemovska and colleagues^25^. Compounds were automatically dispensed into 384-well plates containing 5 μl corresponding media in each well to give a final concentration ranging from to 1 nM to 10 μM. Cells were trypsinized and added to the drug-containing plates at 4000 cells per well. After 72 h incubation at 37 °C and 5% CO_2_, cell viability was measured using CellTiter-Blue fluorescent assay (Promega) as described below. Dose-response curves were generated using the Studies software (Dotmatics Ltd.) as described earlier^25^. Drug Sensitivity Scores (DSS) were calculated for each drug to quantitatively profile the samples^25^,^27^. Briefly, the logistic curve fitting parameters were used to calculate the area under the dose response curve, relative to the total area between 10% threshold and 100% inhibition, which was further normalized by a logarithm of the top response. Differential DSS (dDSS) represents a Z-score from an average DSS value for all cell lines for each drug.

To validate the effects of individual drugs, cells were assayed in 96-well plates in at least three replicas. Cell were plated at concentrations between 0.4–1.3 × 10^4^ cells/well so as to produce confluent wells after 72 hours (4000 cells/well for MCF7 and MCF10A, 5000 cells/well for HCC1937 and SUM229PE, 8000 cells/well for SUM1315MO2, 9000 cells/well for SUM149PT, 11000 cells/well for MDA-MB-231, and 13000 cells/well for MDA-MB-436) in a volume of 100 μl/well of corresponding media. Serial drug dilutions at desired range were added in 100 μl corresponding media. After 72 h the media was replaced with a 10% CellTiter-Blue reagent (Promega) and fluorescence at 520 nm was measured after 3 h using PHERAStar Plus plate reader (BMG Labtech) to estimate relative amounts of metabolically active cells. Dose response curves were plotted either in Excel or GraphPad Prism software.

### Western blot

Cells were prepared with a standard procedure using a modified RIPA buffer (50 mM Tris-HCl pH8.0, 250 mM NaCl, 2 mM EDTA, 1.0% Triton X-100, 0.5% Sodium Deoxycholate, 0.1% SDS, 5 mM NaF and 5 mM Na_3_VO_4_) supplemented with a protease inhibitor tablet (Thermo Scientific). Protein concentrations were determined using the BCA Protein Assay Reagent (Pierce). Protein electrophoresis was carried out using NuPage 4–12% Bis-Tris precast gels (Invitrogen) in the MOPS running buffer according to manufacturer’s instructions, followed by blotting to a nitrocellulose membrane (Millipore). Membranes were incubated overnight at +4 °C in a blocking buffer (1 × TBS, 0.05% Tween 20, 5% milk) with the following primary antibodies: mouse-anti-BRCA1 (OP92, Calbiochem, diluted 1:500), rabbit-anti-53BP1 (ab21083, Abcam, diluted 1:1000), rabbit-anti-MSH2 (ab16833, diluted 1:500), rabbit-anti-MLH1 (ab92312, diluted 1:1000), rabbit-anti-HPRT (#4158, Cell Signiling Technology, diluted 1:1000), rabbit-anti-PARP (#9541, Cell Signiling Technology, diluted 1:1000) and rabbit-anti-Beta Actin (NB600-505, Novus Biologicals, diluted 1:5000). After incubation with fluorescently labeled secondary antibodies (goat-anti-mouse IRDye 800 CW or goat-anti-rabbit IRDye 680 LT, LI-COR) diluted 1:10000, membranes were scanned using a fluorescence scanner Odyssey (LI-COR Biosciences).

### Immunofluorescence

To visualize DNA repair complexes, cells grown on glass coverslips for 36 hours were gamma-irradiated (5 Gy). Six hours later, cells were fixed with 2% paraformaldehyde diluted in PBS containing 1 mM CaCl_2_ and 0.5 mM MgCl_2_ (PBS^++^) for 15 min. After 3 washes with PBS^++^, cells were permeabilized with 0.5% Triton X-100 for 15 min. Then they were blocked for 30 min in an incubation buffer (0.5% BSA, 0.15% glycine, 0.1% Triton X-100 in 1 × PBS). After blocking, the cells were incubated overnight at 4 °C with mouse-anti-phospho-H2A.X (Ser139, ab22551, Abcam, diluted 1:2000) and rabbit-anti-RAD51 (H-92, Santa Cruz Biotechnology, diluted 1:500), or mouse-anti-BRCA1 (OP92, Calbiochem, diluted 1:500) and rabbit-anti-53BP1 (ab21083, Abcam, diluted 1:1000) antibodies. Secondary goat-anti-mouse Alexa488 and goat-anti-rabbit Alexa594 antibodies were applied for 1 hour at room temperature after 3 washes with the incubation buffer. Images were taken using Nikon Eclipse 90i fluorescent microscope and processed with the Nikon NIS-Elements AR software.

### Proteome profiling

Proteome profiling was performed using Human Phospho-Kinase Antibody Array Kit (R&D Systems) according to manufacturer’s instructions. Briefly, cells were grown in 10-cm culture dishes, rinsed with PBS, and lysed. Protein concentration was estimated with BCA Protein Assay Reagent (Pierce) and 300 μg of each cell lysate was added to pre-blocked antibody array membranes for incubation. Membranes were treated with detection antibody cocktail followed by streptavidin-HRP as instructed, and signal was detected using the enhanced chemiluminescence method (ECL, Pierce).

## Additional Information

**How to cite this article**: Gu, Y. *et al*. BRCA1-deficient breast cancer cell lines are resistant to MEK inhibitors and show distinct sensitivities to 6-thioguanine. *Sci. Rep*. **6**, 28217; doi: 10.1038/srep28217 (2016).

## Supplementary Material

Supplementary Information
