# Supplementary material for: BRCA1-deficient breast cancer cell lines are resistant to MEK inhibitors and show distinct sensitivities to 6-thioguanine
Source: Sci Rep. 2016 Jun 17;6:28217. doi: 10.1038/srep28217 (PMC4911578; doi:10.1038/srep28217)
Supplement: Supplementary Information [file srep28217-s1.pdf]

BRCA1-deficient breast cancer cell lines are resistant to MEK inhibitors and show distinct sensitivities to 6-thioguanine.

Yuexi Gu, Mikko Helenius, Kristiina Väänänen, Daria Bulanova, Jani Saarela, Anna Sokolenko, John Martens, Evgeny Imyanitov, and Sergey Kuznetsov

Supplementary Materials

Supplementary Figure S1. Sequencing chromatograms confirm presence of the signature BRCA1 mutations and lack of corresponding wild-type alleles in four BRCA1-mutant cell lines.

Supplementary Figure S2. PARP cleavage in BRCA1-mutant cell lines upon drug treatment.

Supplementary Table T1. Results of the high-throughput screen shown as differential drug sensitivity scores (dDSS).

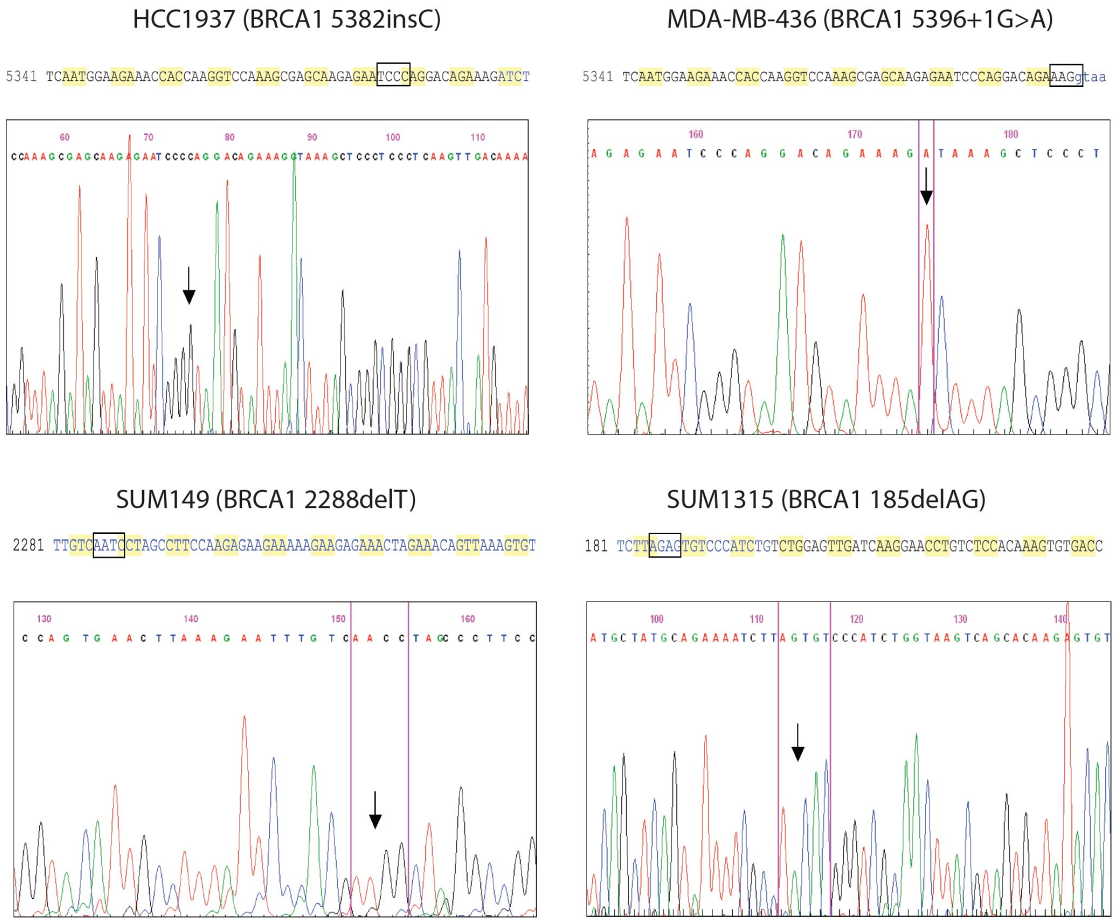

Supplementary Figure S1. **Sequencing chromatograms confirm presence of the signature BRCA1 mutations and lack of corresponding wild-type alleles in four BRCA1-mutant cell lines.** Picks corresponding to mutant nucleotides are marked with arrows. Wild-type BRCA1 sequence encompassing the region of interest is shown at the top of each image and mutant codons are marked with black boxes.

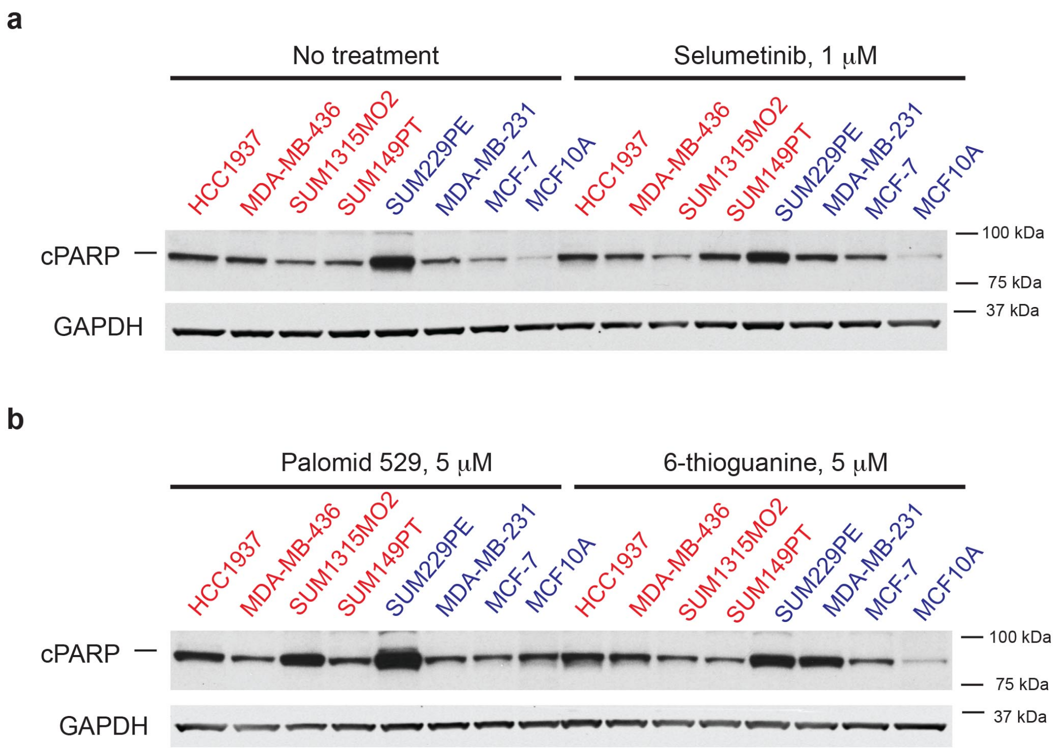

Supplementary Figure S2. **PARP cleavage in BRCA1-mutant cell lines upon drug treatment.** Cells were treated with drugs at indicated concentrations for 18 hours and lysed for Western blotting. Amount of cleaved poly (ADP-ribose) polymerase (cPARP) reflects ongoing apoptosis. GAPDH serves as loading control.

Supplementary Table T1. Results of the high-throughput screen shown as differential drug sensitivity scores (dDSS)*.

		BRCA1-mutant				BRCA1-wildtype					
ID.Drug	Name.Drug	HCC1937	MDA-MB-436	SUM1315MO2	SUM149PT	SUM229PE	MDA-MB-231	MCF-7	MCF-10A	Delta	Molecular target
FIMM003729-001	Pimasertib	-9,715172003	-9,715172003	-9,715172003	-9,715172003	12,84948444	4,048594138	3,333205178	7,072241348	6,825881275	MEK 1/2 inhibitor
FIMM003751-001	Trametinib	-7,341221955	-8,325977935	-6,958447955	-13,3907851	11,80581939	2,959946275	3,140253565	7,680881005	6,396725058	MEK 1/2 inhibitor
FIMM003749-001	BMS-754807	-1,111276139	-6,561887179	-0,597783959	-8,914606429	9,612378231	-2,355546369	17,66779131	-0,330832809	6,148447591	IGF-1R/InsR inhibitor
FIMM000617-002	Fulvestrant	-2,784809989	-2,784809989	-2,784809989	-2,784809989	-2,784809989	-2,784809989	30,63290988	-2,784809989	5,569619978	ER inhibitor
FIMM003754-001	Refametinib	-7,002622151	-8,242354691	-5,463971981	-5,783039831	6,334160619	6,255304409	-2,524564471	10,41647771	5,120344567	MEK 1/2 inhibitor
FIMM023797-001	Floxuridine	2,409238027	-3,184498623	-11,09653277	-11,09653277	13,13085587	-1,473342253	12,6588659	-6,475663763	4,460178937	Antimetabolite, nucleotide analog
FIMM003708-001	Selumetinib	-5,292649443	-5,292649443	-5,292649443	-5,292649443	6,163504278	0,584356177	2,223574468	7,614492928	4,146481963	MEK 1/2 inhibitor
FIMM023798-001	Gemcitabine	0,611681409	-0,446995221	4,318178399	-18,48667654	11,79680024	-5,185326881	9,325754919	0,332288879	4,067379289	Antimetabolite, nucleoside analog
FIMM003786-001	Temsirolimus	3,854659769	-4,484804371	-5,539945951	-13,47138083	10,52939208	6,413513699	12,36115383	-13,47138083	3,958169694	mTOR inhibitor
FIMM003705-001	PD-0332991	-5,31225503	-5,31225503	-5,31225503	-5,31225503	2,8023921	6,49875801	11,36725222	-5,31225503	3,839036825	
FIMM003760-001	MK-2206	5,125092351	-2,271753589	-1,671969719	-7,068874919	5,328875491	-0,994943859	12,89492712	-1,963081419	3,816444333	
FIMM003792-001	Pictilisib	-0,134150198	-2,626180358	-2,507381338	-10,73562336	7,738785113	-3,540364888	12,65730222	-2,078026558	3,694423973	
FIMM003796-001	Patupilone	2,842330588	-2,706040652	3,400522648	-19,26190893	10,35280101	0,671451578	-0,119398102	3,335860058	3,560178636	
FIMM000262-003	Raloxifene	0,409510716	-2,585425584	-1,528933474	-2,585425584	0,366580606	-2,585425584	18,78482266	-2,585425584	3,495138023	
FIMM023815-001	Docetaxel	-1,842264698	-3,402515928	9,265220353	-29,74669778	10,05379665	-2,716659438	0,066910293	6,351522123	3,438892408	
FIMM023819-001	Valrubicin	2,989298601	-3,383119229	-5,596889359	-11,77997026	13,02824315	-2,310476569	3,518125211	-1,119140499	3,279187823	
FIMM003714-001	Danusertib	-2,988563705	-1,920337245	-7,823521145	-7,823521145	4,798844185	0,076439005	5,953947925	2,075449355	3,226170118	
FIMM000491-002	Paclitaxel	-2,309679033	-3,502712123	7,442742768	-18,5570548	9,683906078	-1,529831923	2,677272128	1,625356548	3,114175708	
FIMM000337-002	Vincristine	-5,315782496	1,522743004	1,734780144	-23,02567236	8,637787804	-1,449387036	3,247195564	1,882558484	3,079538704	
FIMM000183-002	Erlotinib	-2,664308166	-1,899146456	0,752126524	-2,664308166	5,022280874	0,648339764	-2,664308166	9,223878724	3,057547799	
FIMM000160-002	Gefitinib	-2,9941122	-2,9941122	4,23395322	-2,9941122	2,90224648	-2,9941122	-2,9941122	14,69731311	2,902833798	
FIMM003710-001	Afatinib	-0,144978517	-4,300783787	4,784182263	-7,134384847	0,581952133	-3,134034797	-0,791755587	14,29790265	2,738516101	
FIMM003783-001	Panobinostat	0,853470053	-4,180553618	-8,102959878	-9,421517688	6,627616973	0,165648463	5,621324503	-1,573223078	2,710341715	
FIMM023812-001	Cytarabine	-1,744759784	-2,916398334	3,713273006	-8,732653964	4,036478256	-4,658921474	8,119573346	3,053804136	2,637733566	
FIMM023832-001	Dactolisib	0,126854388	-1,476735472	-3,680716122	-15,37293694	5,823076978	3,745797738	7,341298868	-6,503904732	2,601567213	
FIMM023795-001	Sirolimus	4,85968998	-5,50632895	-2,64024403	-14,96116892	8,28683852	1,50729532	15,43890427	-14,96116892	2,567967298	
FIMM003768-001	Sunitinib	-2,598627585	-4,513226215	-0,499369695	-4,655744465	5,401979215	4,562521225	3,754508675	-3,820425485	2,474645908	
FIMM003743-001	AZD8055	5,417100672	-2,419412808	-2,788938448	-17,9586654	6,956925522	0,990711492	8,948435992	-7,282626448	2,403361639	
FIMM023818-001	Doxorubicin	2,654073954	-5,357530686	-1,854193676	-3,809000386	8,249238964	-2,867506246	1,645155224	1,968816874	2,248926204	
FIMM003706-001	AT9283	0,4594314	-2,9259463	-7,85364594	-9,18431603	7,61914498	-1,54376143	1,92223876	0,80644139	2,201015925	
FIMM023799-001	Teniposide	2,5839813	-0,05385017	3,58177102	-8,6367024	9,4634122	-3,45745961	4,73915165	-1,99859144	2,1866282	
FIMM000370-002	Vinblastine	-5,786716688	-0,512790868	6,225904322	-18,08992203	7,068922672	-2,035841968	1,797611792	1,890741722	2,180358554	
FIMM000439-003	Fluorouracil	3,184152649	-0,985752981	-0,930639291	-4,799715961	3,138736579	2,955886649	3,210938679	-0,842460131	2,115775444	
FIMM003771-001	YM155	-2,497164548	-5,231490998	-3,950653038	-7,642497368	10,41795658	3,376952113	0,035469653	-5,517972428	2,07810148	
FIMM003735-001	PF-04691502	-1,187814428	-1,112385808	-2,497472198	-8,156755408	4,702234583	0,274256912	6,537763443	-4,018251638	1,874000825	
FIMM003773-001	EMD1214063	-0,625852674	-1,878925354	-3,066969214	-3,066969214	4,758161466	4,489339116	-0,705817924	-1,222927914	1,829688686	
FIMM023800-001	Dactinomycin	-2,477522838	-4,132970038	2,213840393	-3,172460108	4,178410253	-0,983131448	1,196955733	2,645273513	1,759377013	
FIMM023831-001	Bleomycin	-1,956809848	0,067302763	6,465452483	-5,463364708	3,925907953	0,302778243	1,011725353	1,792936173	1,75833693	
FIMM000249-003	Tretinoin	-0,875360103	-0,875360103	-0,875360103	-0,875360103	1,562255387	-0,875360103	7,143425837	-0,875360103	1,738740254	
FIMM100355-001	Sotrastaurin				-0,566350623				1,699051868	1,699051868	
FIMM023811-001	Daunorubicin	2,498153044	-4,149441296	-1,064680036	-6,311802526	8,007477324	-1,396291176	-0,226174236	0,207027504	1,648009854	
FIMM003715-001	Foretinib	-1,114991751	-5,582820591	-1,307751121	-5,189889441	9,173746539	-2,893401481	2,587530119	-2,341163501	1,631677919	
FIMM003748-001	Tacedinaline	-1,142240257	-1,142240257	-1,142240257	-1,142240257	4,301454343	-0,417123367	3,764985963	-1,142240257	1,626769171	
FIMM003797-001	Docetaxel	6,371575521	-2,235249299	8,386640481	-26,22651892	8,762050911	-3,884769439	-1,019598929	2,541727991	1,599852633	
FIMM003787-001	Vorinostat	0,080375623	-2,552522818	-5,667742908	-4,027950338	4,119661153	0,421711203	6,498657253	-4,950821658	1,522301988	
FIMM003772-001	Linsitinib	-1,286314846	-1,286314846	0,095509344	-1,286314846	0,649719814	1,725235844	4,933809784	-1,286314846	1,505612649	
FIMM023802-001	Vinorelbine	4,137708942	-1,488319458	0,024474952	-18,92920613	8,049350932	-2,877034018	0,472626062	0,109136652	1,438519907	
FIMM003779-001	Saracatinib	-0,173105483	-6,358483473	3,141771388	-6,358483473	5,619861558	-0,544962713	3,900299178	-3,226345143	1,43721322	
FIMM003684-002	Irinotecan	-4,090616857	-0,698994777	-0,749756077	-4,090616857	3,325799763	-3,472628777	3,781769813	2,102049653	1,434247613	
FIMM023824-001	Everolimus	1,046152867	-7,400343013	3,444031707	-15,63558881	11,51136338	-2,686915323	13,6701609	-16,89227963	1,400582329	
FIMM003736-001	Rucaparib	-2,718696708	1,012235302	-2,718696708	-2,718696708	6,619980882	0,664817472	0,998487752	-2,718696708	1,391147349	
FIMM003724-001	Decitabine	5,363597644	-3,762872436	-3,762872436	5,112339474	9,529558354	-3,762872436	2,115579644	-2,555820686	1,331611219	
FIMM003781-001	Canertinib	0,803296802	-6,254974808	4,324965672	-1,949753488	1,979354642	-2,022127398	2,397658352	2,914021632	1,317226807	
FIMM003764-001	NVP-AUY922	6,845508357	-7,426549553	-9,639059323	3,826245167	8,422440057	-1,181402853	-7,888319923	5,884014767	1,309183012	
FIMM003744-001	Belinostat	-1,446421218	-4,077600618	-1,438163638	-0,637299817	4,129891593	0,444251123	2,222614253	-1,646388718	1,287592063	
FIMM003734-001	Cediranib	-0,779439314	-3,267742344	2,573294596	-3,267742344	4,591225626	5,255847296	-2,391308784	-2,342882424	1,278220428	
FIMM003722-001	BIIB021	8,546036894	-2,626604336	-8,822212856	-1,504926986	10,16401442	-5,115378406	-0,061000496	0,099372654	1,271752044	
FIMM003755-001	Everolimus	1,210594404	-6,740217056	0,888916174	-15,78331746	7,802197484	-0,920848646	13,87269208	-15,78331746	1,242680867	
FIMM000484-001	Methylprednisolone	-0,771154761	-0,771154761	-0,771154761	0,589255459	-0,771154761	-0,771154761	7,122292149	-0,771154761	1,202206967	
FIMM003713-001	Crizotinib	-1,35946795	-2,26993621	-2,26993621	-2,26993621	5,95627218	0,00107632	0,10826474	-1,26234614	1,200816775	
FIMM003761-001	Alisertib	-0,859291818	-0,147641808	9,670192232	-11,41145222	8,794368132	0,746068722	6,637345252	-11,41145222	1,191582472	
FIMM003777-001	Fingolimod	1,658327373	-5,127245118	1,379387743	-1,069176348	2,403146463	1,363531273	2,456501603	-1,474102048	1,187269323	
FIMM003790-001	Olaparib	-1,871335379	2,473025061	-1,871335379	-1,871335379	1,417297711	-1,871335379	6,922921091	-1,871335379	1,149387011	
FIMM000296-001	Bexarotene	-1,309011366	-1,309011366	-1,309011366	-1,309011366	2,826859534	-1,134726276	4,137105534	-1,309011366	1,130056857	
FIMM023826-001	Clofarabine	3,608882818	-0,513279532	7,332456718	-13,67573908	8,103386828	-2,771663892	-0,737534802	-0,098626012	1,123890531	
FIMM003725-001	Alvocidib	1,740234577	-3,416475773	2,854121377	-2,777518183	2,010013457	0,565526397	1,572330467	0,252870467	1,100185197	
FIMM003728-001	Tipifarnib	7,803113127	-5,088700193	1,820062397	-12,76061724	7,729740207	-3,098866303	12,38170063	-12,76061724	1,062989322	
FIMM003742-001	AZD1152-HQPA	1,950802798	2,091804618	-4,892866003	-9,978957783	7,723999628	1,702185778	4,672455408	-9,978957783	1,029920758	
FIMM100366-001	UCN-01				-9,467480818				1,010743953	1,010743953	
FIMM003719-001	SNS-032	0,823828971	-5,998335339	0,585673671	-5,373549269	5,733781391	-1,339017159	7,008512281	-7,383301259	1,004993813	
FIMM023821-001	Ixabepilone	2,744281856	2,121335006	6,380709216	-11,23214782	6,805152976	-1,340959374	-0,515538734	-1,040113384	0,977135371	
FIMM003753-001	OSI-027	1,019553017	-3,680418763	5,384486377	-7,216434513	0,074781697	5,726043207	4,262744187	-6,305005553	0,939640884	
FIMM000298-003	Celecoxib	-1,224865367	1,830383383	-1,224865367	-1,224865367	4,897091613	-1,224865367	1,001574333	-1,224865367	0,862233803	
FIMM002337-002	BI 2536	-1,436305419	-2,538002229	7,887512241	-18,81331816	5,421858991	-2,893051209	7,751090971	-7,063733069	0,804041421	
FIMM001062-002	Thioguanine	-0,069738731	0,172124129	-0,708790451	-3,403102371	3,949693819	0,812721689	0,770055529	-2,356079011	0,794098007	
FIMM003767-001	Vemurafenib	-0,368040833	-0,368040833	-0,368040833	-0,368040833	4,048449158	-0,368040833	-0,368040833	-0,368040833	0,736081665	
FIMM000274-002	Lapatinib	0,904201297	-3,859111113	-0,838257323	5,188541437	5,563672937	-3,859111113	1,236481517	-0,010084103	0,732739809	
FIMM003775-001	Dasatinib	3,358805523	-16,87940981	0,033719353	-1,157897157	6,485793323	7,800152243	-8,382729757	-3,146063067	0,689288186	
FIMM023804-001	Cladribine	6,511143291	-2,842273619	4,726337931	-11,10779337	12,59366244	-5,451495049	-2,497292299	-2,063311829	0,645390816	
FIMM023814-001	Etoposide	-1,365180799	-0,797563969	1,872554761	-6,780464229	3,716777391	-2,709873919	0,447802141	1,068064481	0,630692523	
FIMM023827-001	Decitabine	4,826669562	-2,524697048	0,633844852	-2,524697048	8,689702442	-2,524697048	-2,524697048	-1,206173768	0,608533644	
FIMM000173-001	Idarubicin	3,500502742	1,373619522	-0,384589958	-9,639808988	6,820698212	-3,122048998	3,630376552	-5,009250778	0,579943747	
FIMM003712-001	Obatoclax	9,864303433	-7,442993307	-2,120407257	-11,95895443	12,22014518	0,643370883	-2,351655837	-8,800986677	0,427718388	
FIMM003778-001	Axitinib	-2,207407468	1,302959973	-2,207407468	-2,207407468	4,551402623	-2,207407468	1,560789143	-2,207407468	0,424344208	
FIMM003709-001	Veliparib	-0,210844695	-0,210844695	-0,210844695	-0,210844695	-0,210844695	-0,210844695	2,319291645	-0,210844695	0,42168939	
FIMM003711-001	Dovitinib	-0,999225337	-0,999225337	-0,999225337	0,257465483	4,563441003	-0,999225337	-0,999225337	-0,999225337	0,391441248	
FIMM003795-001	Seliciclib	0,359259082	-1,025079978	2,057909122	-1,025079978	-1,025079978	4,435732872	-1,025079978	-1,025079978	0,340123234	
FIMM003763-001	Nilotinib	-0,183708595	-0,358168655	-0,358168655	-0,358168655	2,397236355	-0,358168655	-0,358168655	-0,358168655	0,330682598	
FIMM003726-001	MGCD-265	-1,125152258	-1,125152258	-1,125152258	-1,125152258	4,661061412	-1,125152258	-1,125152258	-1,125152258	0,321401159	
FIMM000263-003	Camptothecin	1,0966604	0,52389835	2,29443513	-7,95274399	7,97609892	-10,09304671	4,97712701	-1,60670879	0,313367608	
FIMM000397-003	Dacarbazine	-1,838020604	-1,838020604	-1,838020604	-1,838020604	4,479420696	-1,838020604	-1,838020604	0,317519776	0,280224816	
FIMM003593-002	Exemestane	-0,28286458	-0,28286458	-0,28286458	-0,28286458	1,83297645	-0,28286458	-0,28286458	-0,28286458	0,246095678	
FIMM003738-001	Tivozanib	-2,451576526	-0,072634626	-0,589305916	-2,518190426	4,730273684	0,964598864	-2,281524256	-2,518190426	0,223789467	
FIMM000464-002	Imiquimod	-0,527213178	-0,527213178	-0,527213178	-0,527213178	2,431469072	-0,527213178	-0,527213178	-0,527213178	0,212457384	
FIMM023833-001	Quizartinib	-0,247119801	-0,247119801	-0,247119801	1,009571019	1,180998359	-0,247119801	0,015002229	-0,247119801	0,175440247	
FIMM000172-003	Imatinib	-0,503865063	-0,503865063	-0,503865063	-0,503865063	2,074272028	-0,503865063	-0,503865063	-0,503865063	0,14066921	
FIMM003788-001	Entinostat	1,594675433	-4,324555427	7,589787713	-1,904581657	1,398490533	0,195472273	3,269049903	-4,324555427	0,134614321	
FIMM003723-001	CUDC-101	-2,044525194	-3,295373324	-2,286398224	-0,177922864	0,043175256	-1,151924214	-0,361542764	1,980065726	0,127443501	
FIMM023805-001	Mitomycin C	-1,822241477	-0,069648157	7,500269513	-1,822241477	0,962662473	-1,822241477	3,155684173	-1,822241477	0,118465923	
FIMM003791-001	Masitinib	0,113730695	-1,184060975	-1,184060975	-1,184060975	1,730512425	-0,603523615	0,526787775	-1,184060975	0,117428903	
FIMM100362-001	Midostaurin				-7,780700208				0,113859863	0,113859863	
FIMM003769-001	Vandetanib	4,262349538	-5,169803712	0,565071588	-5,169803712	3,239638468	-0,422852412	1,272981158	-3,728880282	0,090221733	
FIMM023823-001	Plicamycin	2,615676707	-7,382931263	3,201336687	8,200432747	4,249065767	-3,449271683	-2,535056933	2,065848257	0,082646352	
FIMM000490-003	Mitoxantrone	2,39506025	0,72433155	-1,09356921	-12,17670853	6,84190668	-3,15932156	2,277444	-5,72947084	0,05763957	
FIMM003785-001	Tandutinib	-0,982139777	-0,982139777	-0,982139777	-0,982139777	3,149642683	-0,982139777	-0,982139777	-0,982139777	0,050805838	
FIMM000649-002	Methotrexate	7,294291948	1,748223878	-5,552033883	-5,552033883	6,725802178	-5,552033883	4,561373668	-5,552033883	0,04577702	
FIMM001000-002	Mercaptopurine	3,601354722	0,482651772	-2,526351978	-3,344143468	4,695444612	-2,827805728	-3,344143468	1,655835642	0,044832764	
FIMM000556-004	Topotecan	0,740680256	3,003078386	4,168080516	-10,67976109	7,324991306	-8,856593574	4,488496496	-2,864356654	0,023134393	
FIMM000620-003	Tamoxifen	0,038358539	-1,173261501	-1,173261501	1,923192859	-0,402436821	-1,173261501	2,804126619	-1,173261501	0,013791699	
FIMM000060-002	Pilocarpine	0	0	0	0	0	0	0	0	0	
FIMM000076-002	Zoledronic acid	0	0	0	0	0	0	0	0	0	
FIMM000171-001	Anagrelide	0	0	0	0	0	0	0	0	0	
FIMM000176-002	Vatalanib	0	0	0	0	0	0	0	0	0	
FIMM000227-001	Dexamethasone	0	0	0	0	0	0	0	0	0	
FIMM000257-001	Goserelin	0	0	0	0	0	0	0	0	0	
FIMM000290-003	Letrozole	0	0	0	0	0	0	0	0	0	
FIMM000303-003	Anastrozole	0	0	0	0	0	0	0	0	0	
FIMM000326-002	Lomustine	0	0	0	0	0	0	0	0	0	
FIMM000356-003	Altretamine	0	0	0	0	0	0	0	0	0	
FIMM000359-003	Amifostine	0	0	0	0	0	0	0	0	0	
FIMM000360-002	Aminoglutethimide	0	0	0	0	0	0	0	0	0	
FIMM000428-001	Finasteride	0	0	0	0	0	0	0	0	0	
FIMM000521-003	Procarbazine	0	0	0	0	0	0	0	0	0	

		BRCA1-mutant				BRCA1-wildtype					
ID.Drug	Name.Drug	HCC1937	MDA-MB-436	SUM1315MO2	SUM149PT	SUM229PE	MDA-MB-231	MCF-7	MCF-10A	Delta	Molecular target
FIMM000523-001	Prednisone	0	0	0	0	0	0	0	0	0	
FIMM000560-003	Temozolomide	0	0	0	0	0	0	0	0	0	
FIMM000618-003	Megestrol acetate	0	0	0	0	0	0	0	0	0	
FIMM000774-002	Aminolevulinic acid	0	0	0	0	0	0	0	0	0	
FIMM000983-002	Hydroxyurea	0	0	0	0	0	0	0	0	0	
FIMM001171-002	Carmustine	0	0	0	0	0	0	0	0	0	
FIMM001217-002	Thiotepa	0	0	0	0	0	0	0	0	0	
FIMM001244-001	Tacrolimus	0	0	0	0	0	0	0	0	0	
FIMM003716-001	Abiraterone	0	0	0	0	0	0	0	0	0	
FIMM003733-001	Motesanib	0	0	0	0	0	0	0	0	0	
FIMM003737-001	Tarenflurbil	0	0	0	0	0	0	0	0	0	
FIMM003745-001	Bimatoprost	0	0	0	0	0	0	0	0	0	
FIMM003747-001	Bryostatin 1	0	0	0	0	0	0	0	0	0	
FIMM003752-001	Erismodegib	0	0	0	0	0	0	0	0	0	
FIMM003757-001	Fasudil	0	0	0	0	0	0	0	0	0	
FIMM003762-001	Nelarabine	0	0	0	0	0	0	0	0	0	
FIMM003776-001	Tofacitinib	0	0	0	0	0	0	0	0	0	
FIMM003782-001	Lenalidomide	0	0	0	0	0	0	0	0	0	
FIMM003784-001	Pazopanib	0	0	0	0	0	0	0	0	0	
FIMM003793-001	Vismodegib	0	0	0	0	0	0	0	0	0	
FIMM023801-001	Streptozocin	0	0	0	0	0	0	0	0	0	
FIMM023806-001	Carboplatin	0	0	0	0	0	0	0	0	0	
FIMM023810-001	Uracil mustard	0	0	0	0	0	0	0	0	0	
FIMM023817-001	Capecitabine	0	0	0	0	0	0	0	0	0	
FIMM003770-001	XL147	-0,001445403	-0,001445403	-0,001445403	-0,001445403	-0,001445403	-0,001445403	-0,001445403	-0,001445403	-0,001445403	
FIMM000403-003	Cyclophosphamide	-0,002862163	-0,002862163	-0,002862163	-0,002862163	-0,002862163	-0,002862163	-0,002862163	-0,002862163	-0,002862163	
FIMM023813-001	Dexrazoxone	-0,004404883	-0,004404883	-0,004404883	-0,004404883	-0,004404883	-0,004404883	-0,004404883	-0,004404883	-0,004404883	
FIMM023794-001	Pentostatin	-0,052536289	-0,052536289	-0,052536289	0,577899181	-0,052536289	-0,052536289	-0,052536289	-0,052536289	-0,052536289	
FIMM000463-003	Ifosfamide	-0,432848765	-0,432848765	-0,432848765	3,312230855	-0,432848765	-0,432848765	1,016256795	-0,432848765	-0,070572375	
FIMM000822-002	Mitotane	-0,086036097	-0,086036097	-0,086036097	-0,086036097	-0,086036097	-0,086036097	-0,086036097	-0,086036097	-0,086036097	
FIMM000773-001	Nilutamide	-0,104724235	-0,104724235	-0,104724235	1,151966585	-0,104724235	-0,104724235	-0,104724235	-0,104724235	-0,104724235	
FIMM003759-001	Iniparib	-0,104724235	-0,104724235	-0,104724235	1,151966585	-0,104724235	-0,104724235	-0,104724235	-0,104724235	-0,104724235	
FIMM003766-001	Plerixafor	-0,104731326	-0,104731326	-0,104731326	1,152044584	-0,104731326	-0,104731326	-0,104731326	-0,104731326	-0,104731326	
FIMM003756-001	Ruxolitinib	-0,115978852	-0,115978852	-0,115978852	1,140711968	-0,115978852	-0,115978852	-0,115978852	-0,115978852	-0,115978852	
FIMM000862-002	Busulfan	-0,731111621	-0,731111621	-0,731111621	-0,731111621	1,690629259	-0,731111621	-0,731111621	-0,731111621	-0,125676401	
FIMM001220-002	Pipobroman	-0,129383898	-0,129383898	-0,129383898	-0,129383898	-0,129383898	-0,129383898	-0,129383898	-0,129383898	-0,129383898	
FIMM100345-001	Prima-1 Met	-0,143885254	-0,143885254	-0,143885254	1,582737796	-0,143885254	-0,143885254	-0,143885254	-0,143885254	-0,143885254	
FIMM000469-001	Levamisole	-0,14886287	-0,14886287	-0,14886287	1,49529714	-0,14886287	-0,14886287	-0,14886287	-0,14886287	-0,14886287	
FIMM000519-001	Prednisolone	-0,174775003	-0,174775003	-0,174775003	1,922525028	-0,174775003	-0,174775003	-0,174775003	-0,174775003	-0,174775003	
FIMM023829-001	Nelarabine	-0,189215809	-0,189215809	-0,189215809	2,081373901	-0,189215809	-0,189215809	-0,189215809	-0,189215809	-0,189215809	
FIMM003750-001	CAL-101	0,99913361	-1,6605232	-1,6605232	-1,6605232	-1,6605232	-0,80116716	3,33190377	-1,6605232	-0,197577448	
FIMM003739-001	MK-1775	7,715780717	-2,545758923	0,501680397	-13,50287567	7,813808507	-1,662431303	2,084630637	-9,052851693	-0,204210963	
FIMM000390-003	Thalidomide	-0,204367977	-0,204367977	-0,204367977	-0,204367977	-0,204367977	-0,204367977	-0,204367977	-0,204367977	-0,204367977	
FIMM003727-001	Amonafide	-0,404571433	-2,645751533	-2,879611033	-0,399540863	5,909347147	-5,307277903	-1,114464483	-0,342829263	-0,213806126	
FIMM001003-002	Methoxsalen	-0,231593399	-0,231593399	-0,231593399	2,547527391	-0,231593399	-0,231593399	-0,231593399	-0,231593399	-0,231593399	
FIMM023803-001	Triethylenemelamine	-0,241654269	-0,241654269	-0,241654269	-0,241654269	-0,241654269	-0,241654269	-0,241654269	-0,241654269	-0,241654269	
FIMM003741-001	AZD7762	5,874437741	-1,166342799	-1,588871539	-2,062976979	9,086460021	-3,985262679	2,249832631	-8,327138819	-0,244027212	
FIMM100356-001	XL765				0,938362113				-0,318328708	-0,318328708	
FIMM003765-001	2-methoxyestradiol	1,447347808	-6,698241912	7,270182388	-7,392986542	6,997051948	-0,963525652	-0,464657492	-6,936596572	-0,341931942	
FIMM000377-003	Chlorambucil	-0,374948725	-0,374948725	-0,374948725	-0,374948725	-0,374948725	-0,374948725	-0,374948725	-0,374948725	-0,374948725	
FIMM000446-001	Flutamide	-0,432549614	-0,432549614	-0,432549614	1,562271386	-0,432549614	-0,432549614	-0,432549614	-0,432549614	-0,432549614	
FIMM003721-001	Serdemetan	3,842403083	0,514396443	-5,290322348	-5,290322348	8,580124493	-0,485233348	-4,801840918	-5,290322348	-0,49931803	
FIMM000833-002	Allopurinol	-0,192106765	-0,862056005	1,595769775	-0,862056005	0,544579245	-0,862056005	-0,862056005	-0,862056005	-0,510397193	
FIMM003717-001	Tanespimycin	8,831100803	-5,049708748	2,396190243	-0,215879637	6,473837163	-7,973245768	-3,366086058	2,726883173	-0,534652873	
FIMM003746-001	Doramapimod	-0,572340714	-0,572340714	5,039057036	0,684350106	-0,572340714	-0,572340714	-0,572340714	-0,572340714	-0,572340714	
FIMM023828-001	Fludarabine	-2,01140388	-2,01140388	3,36459446	-2,01140388	1,4206238	-0,53159623	-1,29017041	-2,01140388	-0,60313668	
FIMM003774-001	Lestaurtinib	1,136301538	-0,927759132	4,900661478	-3,541355352	3,537475088	-2,731409612	-2,470471312	-0,972816752	-0,659305647	
FIMM003730-001	Ponatinib	-2,72455227	-4,70004854	4,89813273	-2,06288013	4,95582898	1,44913077	-7,77649864	-1,37730619	-0,68721127	
FIMM003718-001	Alvespimycin	5,715843542	-3,241971528	-4,184817198	0,522442302	6,103391132	-6,991661988	-4,947364448	2,797599042	-0,759509066	
FIMM100347-001	Gandotinib			3,190315344	0,031903214		-5,099008926	3,819159664	-1,067026196	-0,782291819	
FIMM003707-001	Navitoclax	-2,259660723	-2,259660723	-2,259660723	-2,259660723	-2,259660723	3,528312107	-2,259660723	-2,259660723	-0,812667516	
FIMM003794-001	Sorafenib	3,676914088	-3,274974173	3,777937068	-0,830103723	-1,614146793	4,249088338	-2,335030983	-3,784150933	-0,871060093	
FIMM100352-001	AZ 3146				3,231630315				-1,077210105	-1,077210105	
FIMM003720-001	ABT-751	2,379931457	0,204345317	5,095158527	-9,092116573	3,975140387	-3,229086063	-3,159865593	-1,918222713	-1,083008496	
FIMM023825-001	Azacitidine	1,717298768	1,324137778	10,62145304	-2,543106432	-2,543106432	-0,242883292	-2,543106432	0,449650718	-1,219861359	
FIMM003789-001	Enzastaurin	3,455188384	2,434600264	7,167757174	-4,338800586	8,005840894	-4,338800586	-4,338800586	-4,338800586	-1,252640216	
FIMM100346-001	Carfilzomib	2,808835921	-5,840015969	-4,660695219	1,120010461	7,476846371	0,221253021	-1,482491989	-11,23254017	-1,254233192	Proteasome inhibitor
FIMM023822-001	Melphalan	2,937992609	1,159902859	2,085956829	-2,402667591	1,760126269	-2,402667591	-2,402667591	-2,402667591	-1,361969126	
FIMM100365-001	XAV-939				-1,497315553				-1,497315553	-1,497315553	
FIMM100364-001	Regorafenib				-1,808632283				-1,808632283	-1,808632283	
FIMM003758-001	Indibulin	5,452581316	3,778497246	6,943407556	-12,26460516	9,147319996	-6,787163174	1,306325616	-12,26460516	-2,149530682	
FIMM003740-001	PF-00477736	1,491729877	-0,128725483	4,690399167	-1,073588593	-1,059772113	-2,223066253	1,617949347	-7,584975693	-2,312466178	
FIMM100367-001	Ruboxistaurin				-2,318760048				-2,509438878	-2,509438878	
FIMM100354-001	S-trityl-L-cysteine				-3,112326743				-3,065414533	-3,065414533	
FIMM023830-001	Bortezomib	2,832396061	-4,945013359	4,915796951	2,435899761	8,589206171	1,325788211	-6,910191029	-21,96409844	-4,739823772	Proteasome inhibitor

* - DSS were calculated for each drug and normalized by the logarithm of the top response. Positive dDSS values indicate sensitivity relative to the average from all cell lines, negative – relative resistance, respectively. "Delta" column shows the difference between average dDSS score for all BRCA1-mutant cell lines and the average for all BRCA1-wildtype cells (positive Delta indicates that BRCA1-mutant cells are mostly resistant relative to control cell lines). Results for MEK1/2 inhibitors are highlighted in red.
